# Synthesis, ^18^F-Radiolabelling and Biological Characterization of Novel Fluoroalkylated Triazine Derivatives for *in Vivo* Imaging of Phosphodiesterase 2A in Brain via Positron Emission Tomography

**DOI:** 10.3390/molecules20069591

**Published:** 2015-05-26

**Authors:** Susann Schröder, Barbara Wenzel, Winnie Deuther-Conrad, Rodrigo Teodoro, Ute Egerland, Mathias Kranz, Matthias Scheunemann, Norbert Höfgen, Jörg Steinbach, Peter Brust

**Affiliations:** 1 Department of Neuroradiopharmaceuticals, Institute of Radiopharmaceutical Cancer Research, Helmholtz-Zentrum Dresden-Rossendorf, Permoserstraße 15, Leipzig 04318, Germany; E-Mails: b.wenzel@hzdr.de (B.W.); w.deuther-conrad@hzdr.de (W.D.-C.); r.teodoro@hzdr.de (R.T.); m.kranz@hzdr.de (M.K.); m.scheunemann@hzdr.de (M.S.); j.steinbach@hzdr.de (J.S.); p.brust@hzdr.de (P.B.); 2 BioCrea GmbH, Meissner Str. 191, Radebeul 01445, Germany; E-Mails: Ute.Egerland@biocrea.com (U.E.); Norbert.Hoefgen@biocrea.com (N.H.)

**Keywords:** PDE2A, Alzheimer’s disease, PET imaging in brain, micellar HPLC

## Abstract

Phosphodiesterase 2A (PDE2A) is highly and specifically expressed in particular brain regions that are affected by neurological disorders and in certain tumors. Development of a specific PDE2A radioligand would enable molecular imaging of the PDE2A protein via positron emission tomography (PET). Herein we report on the syntheses of three novel fluoroalkylated triazine derivatives (**TA2**–**4**) and on the evaluation of their effect on the enzymatic activity of human PDE2A. The most potent PDE2A inhibitors were ^18^F-radiolabelled ([^18^F]**TA3** and [^18^F]**TA4**) and investigated regarding their potential as PET radioligands for imaging of PDE2A in mouse brain. *In vitro* autoradiography on rat brain displayed region-specific distribution of [^18^F]**TA3** and [^18^F]**TA4**, which is consistent with the expression pattern of PDE2A protein. Metabolism studies of both [^18^F]**TA3** and [^18^F]**TA4** in mice showed a significant accumulation of two major radiometabolites of each radioligand in brain as investigated by micellar radio-chromatography. Small-animal PET/MR studies in mice using [^18^F]**TA3** revealed a constantly increasing uptake of activity in the non-target region cerebellum, which may be caused by the accumulation of brain penetrating radiometabolites. Hence, [^18^F]**TA3** and [^18^F]**TA4** are exclusively suitable for *in vitro* investigation of PDE2A. Nevertheless, further structural modification of these promising radioligands might result in metabolically stable derivatives.

## 1. Introduction

Phosphodiesterases (PDEs) are a class of intracellular enzymes consisting of 11 families and 21 isoforms [1,2,3,4]. The PDE family subtypes differ in their three-dimensional structure, kinetic and regulatory properties, cellular expression, intracellular location, inhibitor sensitivities, substrate selectivity and distribution within the organism. Due to hydrolysis of the cyclic nucleotides, PDEs affect the second messenger signaling cascades of cyclic adenosine monophosphate (cAMP) and/or cyclic guanosine monophosphate (cGMP) [1,2]. Therefore, pharmacological inhibition of PDEs has the potential to enhance cyclic nucleotide signaling and can provide a strategy for the prevention or treatment of various diseases [2,5,6,7,8].

Phosphodiesterase 2A (PDE2A) is a dual-substrate specific enzyme degrading both nucleotides cAMP and cGMP. The PDE2A protein is highly and specifically expressed in particular brain regions such as striatum, cortex, hippocampus, substantia nigra, amygdala, and in olfactory neurons [9,10,11,12].

The specific distribution of the PDE2A protein in brain indicates a modulation of important neuronal functions associated to learning and memory [13,14,15]. Inhibition of PDE2A activity in brain is suggested to improve neuronal plasticity and memory formation due to increased cGMP levels in active synapses [16,17]. Hence, PDE2A inhibitors might be promising compounds regarding drug development for treatment of neurodegenerative disorders such as Alzheimer’s disease (AD) [3,12]. In addition, this enzyme is highly expressed in certain tumors such as malignant melanoma and mammary carcinoma [18,19], and it is assumed that PDE2A activity is related to highly proliferative processes [20,21].

**Figure 1:**
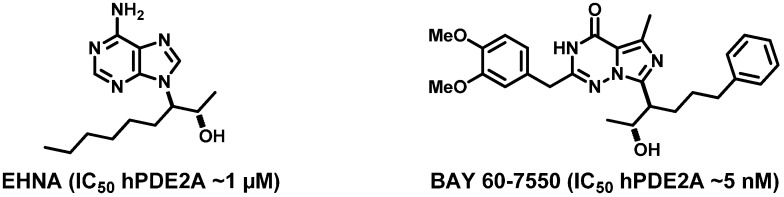
Most common PDE2A inhibitors: EHNA and BAY 60-7550 [2,16,17,22,23,24,25,26,27,28].

The most common PDE2A inhibitors, shown in Figure 1, are *erythro*-9-(2-hydroxy-3-nonyl)-adenine (EHNA) [2,16,22,23] and BAY 60–7550 [2,16,17,24,25,26,27,28].

**Figure 2:**
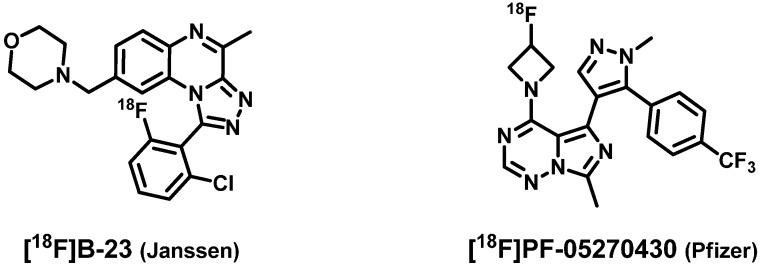
PDE2A radioligands developed by Janssen ([^18^F]B-23) and Pfizer ([^18^F]PF-05270430) [3,36,37].

Besides further PDE2A inhibitors developed for treatment of neurological disorders [3,29,30,31,32,33,34,35], two PDE2A radioligands for molecular imaging of this protein in the brain via positron emission tomography (PET) have been reported to date (Figure 2).

The PET ligand [^18^F]B-23 developed by Janssen [3,36] is highly affine towards the PDE2A protein but also for PDE10A (IC_50_ hPDE2A = 1 nM; IC_50_ rPDE10A = 11 nM). In microPET imaging studies with rats the highest accumulation of [^18^F]B-23 has been observed in the striatum, however, radio-metabolites have been detected in the brain (at 2 min p.i.: 4%; at 10 min p.i.: 10% of total activity). The distribution pattern of PDE2A and PDE10A is comparable: e.g., both proteins are highly expressed in the caudate nucleus [9]. Thus, the reported uptake in the striatum may be caused by binding of [^18^F]B-23 to both enzymes in this brain region. Hence, to develop appropriate radioligands for PET imaging of PDE2A, high selectivity toward this protein is of major importance. The radioligand [^18^F]PF-05270430 published by Pfizer [3,37] is a highly affine and selective PDE2A inhibitor (IC_50_ hPDE2A = 0.5 nM; IC_50_ hPDE10A = 3.0 µM) with good brain uptake. In PET studies on monkeys a rapid and high uptake of [^18^F]PF-05270430 in the striatum has been reported. Supplementary data of [^18^F]PF-05270430 concerning dosimetry and radiometabolite analysis have not been published yet.

**Figure 3:**
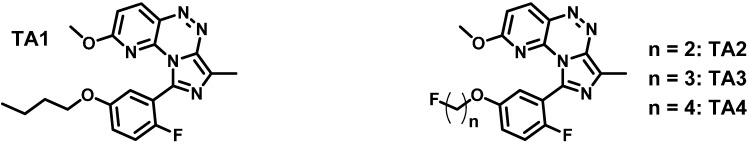
Triazine lead compound **TA1** and the novel fluoroalkylated derivatives **TA2**–**4** as PDE2A ligands.

The goal of our work is the imaging of the PDE2A protein in brain via PET that may enable early diagnostics of related diseases. Furthermore, specific PDE2A radioligands may also be used for the pharmacological characterization and evaluation of novel PDE2A inhibitors as therapeutics. Herein, we present the development of three novel fluoroalkylated derivatives (**TA2**–**4**) as PDE2A ligands starting from a triazine lead compound (**TA1**) [38] (Figure 3).

Out of this series of derivatives, the two most suitable PDE2A inhibitors were ^18^F-radiolabelled and investigated regarding their (i) *in vitro* properties using rat brain slices; (ii) metabolic stability in mice; and (iii) *in vivo* imaging potential using small-animal PET in mice.

## 2. Results and Discussion

### 2.1. Synthesis and in Vitro Binding

**Scheme 1:**
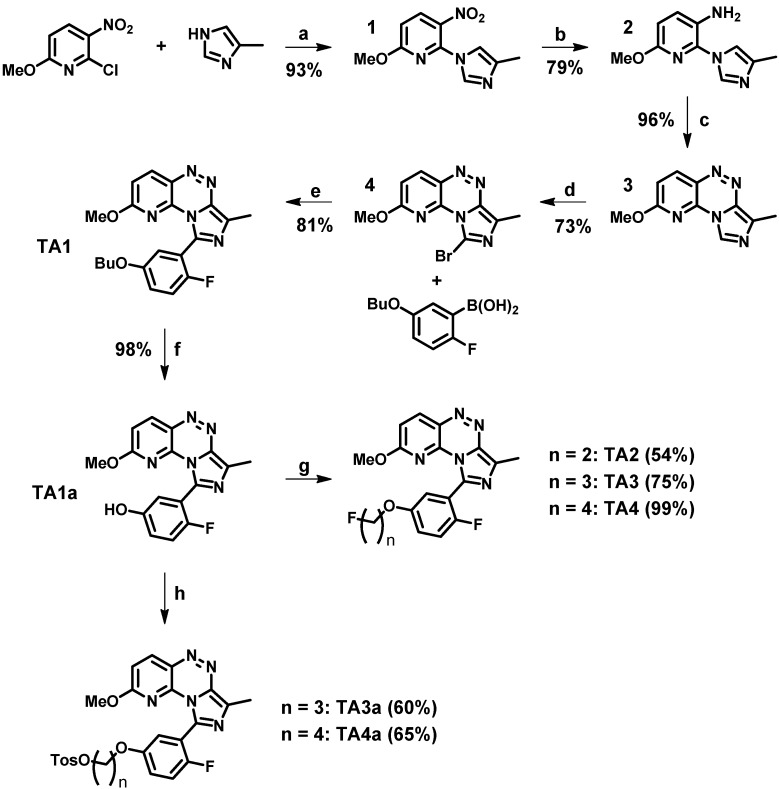
Syntheses of the triazine lead compound **TA1**, the phenolic intermediate **TA1a**, the novel fluoroalkylated PDE2A ligands **TA2**–**4** and the tosylate precursors **TA3a** and **TA4a**.

The novel fluoroalkylated derivatives **TA2**–**4** presented as PDE2A ligands were developed starting from the triazine lead compound **TA1** [38]. The structure of **TA1** (Figure 3) contains a fluorine atom on the benzene ring. In terms of a planned ^18^F-radiolabelling, this position is not activated for a nucleophilic aromatic substitution of a leaving group by [^18^F]fluoride due to the enhanced electron density [39]. Therefore, we favored the introduction of a second fluorine atom in the phenolic ether group enabling a nucleophilic ^18^F-radiolabelling at the alkyl side chain. The five steps synthesis of the lead compound **TA1** has already been reported [38] and was partly optimized in this study (Scheme 1).

*Reagents and Conditions*: (**a**) 3 eq. TEA, 10 mol % DMAP, CHCl_3_, 0 °C to RT, overnight; (**b**) Pd(C)/H_2_, EtOH, RT, overnight; (**c**) 1.5 eq NaNO_2_, H_2_O/CH_3_COOH, ≤5 °C, 30 min; (**d**) 1.5 eq *N*-bromosuccinimide (NBS), CH_2_Cl_2_, ≤5 °C to RT, overnight; (**e**) 1 eq 5-butoxy-2-fluorophenyl boronic acid, 0.05 eq [(Ph_3_)P]_4_Pd(0), 3 eq K_2_CO_3_, dioxan/H_2_O, 90 °C, 5 h; (**f**) 3.05 eq BBr_3_ (1 M in CH_2_Cl_2_), CH_2_Cl_2_, ≤5 °C, 2 h; (**g**) *n* = 2: F-(CH_2_)_2_-I, *n* = 3: F-(CH_2_)_3_-I, *n* = 4: F-(CH_2_)_4_-Br, 3 eq K_2_CO_3_, MeCN, 70–80 °C, 5 h; (**h**) *n* = 3: TosO-(CH_2_)_3_-OTos, *n* = 4: TosO-(CH_2_)_4_-OTos, 4 eq K_2_CO_3_, MeCN, 60–70 °C, 5–10 h.

The first step relates to the coupling reaction between the substituted 2-chloropyridine and the 4-methylimidazole component to afford **1**. By using triethylamine (TEA) as base in the presence of 4-(dimethylamino)pyridine (DMAP) as catalyst instead of potassium carbonate, and replacing *N*,*N*-dimethylformamide (DMF) by chloroform as solvent, **1** was obtained isomerically pure and with a 20% higher yield (93% *vs.* 71% [38]). Notably, a synthesis of compound **1** [38] resulting in an inseparable 4:1 mixture of the imidazole regioisomers has also been published by Malamas *et al.* [40]. For the reduction of the nitro group to the corresponding amine, milder reaction conditions (e.g., temperature and pressure) were used, affording **2** in similar yields (79% *vs.* 81% [38]). Afterwards, a diazotization was performed followed by an intramolecular cyclisation (azo coupling) to get the triazine basic structure **3**. By washing the precipitate instead of recrystallization or column chromatography, **3** was obtained in comparable yields (96% *vs.* 93% [38]). The bromination at the imidazole site has been reported for the corresponding 4-methoxy compound [38]. The 2-methoxy-bromo derivative **4** was obtained in similar yields according to the literature (73% *vs.* 76% [38]). Finally, the Suzuki coupling with the 5-butoxy-2-fluorophenyl boronic acid was performed as previously reported [38] affording the lead compound **TA1** in 81% yield.

The subsequent cleavage of the butoxy group by boron tribromide resulted in the phenol compound **TA1a** in 98% yield. Notably, the butoxy group was selectively cleaved while the 2-methoxy function remained stable, even in the presence of a large excess of boron tribromide (up to 10 eq.). The novel derivatives **TA2**, **TA3** and **TA4** were successfully synthesized in 54%, 75% and 99% yield, respectively, using the phenolic intermediate **TA1a** and appropriate fluoroalkyl halides (Scheme 1).

The novel fluoroalkylated derivatives **TA2**–**4** were evaluated in an enzyme assay [38] to determine their inhibitory potencies for the human recombinant PDE2A and PDE10A proteins. The IC_50_ values obtained by this assay represent relative measures of the respective target affinity of the compounds. We have previously shown for a specific PDE10A radioligand that the target affinity is within the same order of magnitude as the inhibitory potency of the corresponding non-radioactive reference compound [41].

As mentioned, only ligands with high affinity and selectivity are suitable for PET imaging of PDE2A due to the comparable distribution pattern of PDE2A and PDE10A in the brain [9]. Table 1 summarizes the IC_50_ values of the lead compound **TA1** and the novel fluoroalkylated derivatives **TA2**–**4** for the inhibition of human PDE2A and human PDE10A.

**Table 1:** IC_50_ values of the lead compound **TA1** and the novel fluoroalkylated derivatives **TA2**–**4** for the inhibition of human PDE2A and human PDE10A.

Ligand	IC_50_ hPDE2A	IC_50_ hPDE10A	Selectivity Ratio PDE10A/PDE2A
**TA1** (lead)	4.5 nM	670 nM	148.9
**TA2** (2-fluoroethyl)	10.4 nM	77 nM	7.4
**TA3** (3-fluoropropyl)	**11.4 nM**	**318 nM**	**27.9**
**TA4** (4-fluorobutyl)	**7.3 nM**	**913 nM**	**125.1**

Compared to the lead compound **TA1** the affinity and selectivity of **TA2** and **TA3** are slightly lower. Besides its high potency, **TA4** shows the highest selectivity of all tested fluoroalkylated derivatives. These findings can be explained by the recently reported binding-induced hydrophobic pocket (H-pocket) in the active center of the PDE2A protein [3,42]. This mechanism suggests that hydrophobic interactions between the propylphenyl group of BAY 60–7550 and the H-pocket might be responsible for the high PDE2A selectivity of this compound [42]. Accordingly, the strength of hydrophobic interactions with the H-pocket may be related to the increased chain lengths within the novel derivatives **TA2**–**4**.

Thus, the ligand **TA3** and the very promising derivative **TA4** were selected as candidates for ^18^F-radiolabelling. The corresponding tosylate precursors **TA3a** and **TA4a**, necessary for a one-step nucleophilic ^18^F-radiolabelling strategy, were synthesized with yields of 60%–65% by reaction of propane-1,3-diyl bis(4-methyl-benzenesulfonate) or butane-1,4-diyl bis(4-methyl-benzenesulfonate) with the phenolic intermediate **TA1a** (see Scheme 1).

### 2.2. Radiochemistry, Lipophilicity and in Vitro Stability

The novel PDE2A radioligands [^18^F]**TA3** and [^18^F]**TA4** were prepared in a one-step radiosynthesis by nucleophilic substitution of the tosylate group of the precursors **TA3a** and **TA4a** with the anhydrous K^+^/[^18^F]F^−^/K_2.2.2_-carbonate complex in acetonitrile (for the drying procedure see Section 3.4.1.).

**Figure 4:**
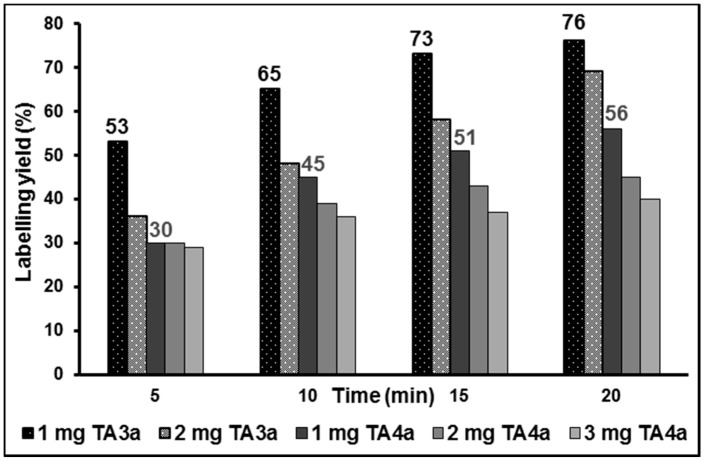
Optimization of the labelling yields for aliphatic ^18^F-radiolabelling of the tosylate precursors **TA3a** and **TA4a** by varying the amount of precursor and the reaction time (in MeCN at 80 °C, conventional heating).

Optimization of the aliphatic radiolabelling was performed by varying the amount of precursor (1–3 mg) and reaction time (up to 20 min) under conventional heating at 80 °C in acetonitrile. As shown in Figure 4, the highest labelling yields were achieved by using 1 mg of the corresponding tosylate precursor **TA3a** or **TA4a**. Accordingly, an increase of the amount of tosylate precursor **TA3a** resulted in an unexpected decrease of the labelling yield, which is more pronounced at the early time points. Independent of the amount of precursor **TA3a**, only a single ^18^F-side product was detected with 4% of total activity at 15 min reaction time. Both precursors were stable under the reaction conditions over 20 min, proved by HPLC.

**Scheme 2:**
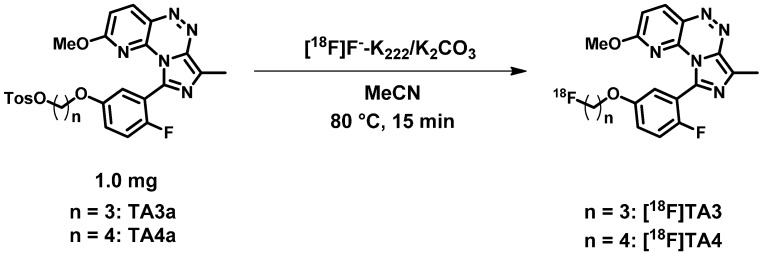
Nucleophilic ^18^F-radiolabelling of tosylate precursors **TA3a** and **TA4a** to generate the novel PDE2A radioligands [^18^F]**TA3** and [^18^F]**TA4**.

A time-dependent increase of the labelling yields was observed, however, 73% for [^18^F]**TA3** and 51% for [^18^F]**TA4** after 15 min reaction time are sufficiently high for further experiments. Thus, manual radiosyntheses of [^18^F]**TA3** and [^18^F]**TA4** were performed in acetonitrile at 80 °C for 15 min under no-carrier-added (n.c.a.) conditions using 1 mg of each tosylate precursor **TA3a** or **TA4a** (Scheme 2).

**Figure 5:**
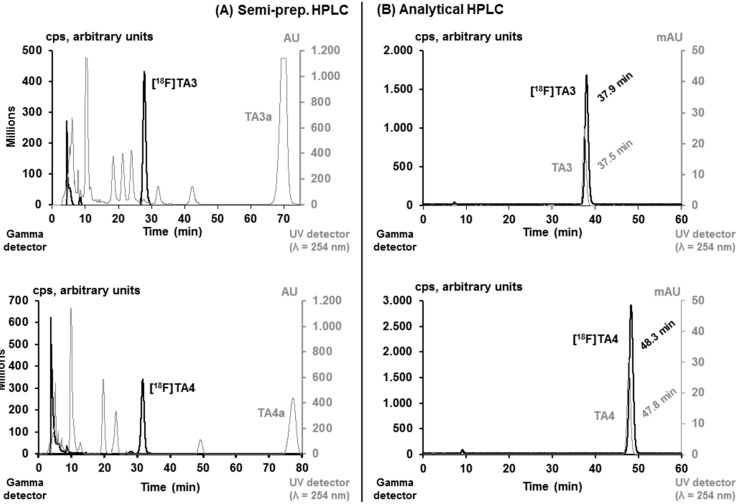
(**A**) HPLC profile of the crude reaction mixtures for semi-preparative isolation of [^18^F]**TA3** and [^18^F]**TA4** (column: Reprosil-Pur C18-AQ, 250 × 10 mm, particle size: 10 µm; eluent: 50% MeCN/20 mM NH_4_OAc_aq._; flow: 3 mL/min for [^18^F]**TA3**, 3.5 mL/min for [^18^F]**TA4**); (**B**) Analytical HPLC profile of the formulated radioligands [^18^F]**TA3** and [^18^F]**TA4** spiked with the non-radioactive reference compounds **TA3** and **TA4** (column: Reprosil-Pur C18-AQ, 250 × 4.6 mm, particle size: 5 µm; eluent: 44% MeCN/20 mM NH_4_OAc_aq._; flow: 1 mL/min).

[^18^F]**TA3** and [^18^F]**TA4** were isolated by semi-preparative HPLC (system **A**; *t_R_* = 27–33 min, see Figure 5), purified using solid phase extraction on a pre-conditioned RP cartridge and eluted with absolute ethanol. The solvent was evaporated at 70 °C and the radioligands were formulated in sterile isotonic saline containing 10% of ethanol (*v*/*v*) for better solubility. Aliquots of the final products were spiked with the related non-radioactive reference compounds **TA3** and **TA4** to verify the identities of [^18^F]**TA3** and [^18^F]**TA4** (Figure 5) by analytical HPLC (system **B**).

The radioligands were synthesized with moderate to high labelling yields of 72.5% ± 6.4% for [^18^F]**TA3** (*n* = 8) and 41.5% ± 9.6% for [^18^F]**TA4** (*n* = 4), radiochemical yields of 48.7% ± 8.5% for [^18^F]**TA3** (*n* = 4) and 25.4% ± 3.9% for [^18^F]**TA4** (*n* = 4), specific activities (EOS) of 60.4 ± 11.6 GBq/µmol for [^18^F]**TA3** (*n* = 3) and 77.1 ± 23.8 GBq/µmol for [^18^F]**TA4** (*n* = 3), and high radiochemical purities of ≥99%. The decreased labelling yield of [^18^F]**TA4** in comparison with [^18^F]**TA3** could be a result of the additional CH_2_ group. The electron withdrawing effect of the phenolic oxygen on the alkyl side chain might be slightly reduced due to the longer distance to the carbon atom that is attacked by the nucleophilic [^18^F]F^−^. For example, a similar effect has been observed for [^18^F]fluoroalkoxy derivatives of harmine (7-methoxy-1-methyl-9H-β-carboline) where a decreased radiochemical yield of the 7-(3-[^18^F]fluoropropoxy) analogue in comparison to the 7-(2-[^18^F]fluoroethoxy) derivative has been reported [43].

To estimate the lipophilicity of [^18^F]**TA3** and [^18^F]**TA4**, the distribution coefficients were determined by partitioning between *n*-octanol and phosphate buffered saline (PBS, pH 7.4) at ambient temperature using the conventional shake-flask method. LogD values of 3.37 ± 0.14 for [^18^F]**TA3** and 2.99 ± 0.15 for [^18^F]**TA4** were obtained, indicating a high to moderate lipophilicity regarding passive transport across the blood brain barrier. *In vitro* stability of each radioligand was investigated in phosphate buffered saline (PBS, pH 7.4), *n*-octanol and pig plasma after 60 min incubation at 37 °C. [^18^F]**TA3** and [^18^F]**TA4** proved to be stable in all media tested *in vitro*, and no defluorination or degradation was observed by radio-TLC or analytical radio-HPLC.

Due to the higher metabolic stability of [^18^F]**TA3** *in vivo* compared to that of [^18^F]**TA4** as described below in Section 2.4., the automated radiosynthesis was performed only of [^18^F]**TA3**. The conditions for the manual radiosynthesis of [^18^F]**TA3** (^18^F-labelling: 1 mg of tosylate precursor **TA3a** in MeCN, 80 °C, 15 min) were transferred to an automated process using a TRACERlab™ FX F-N synthesis module. After automated isolation of [^18^F]**TA3** by semi-preparative HPLC (system **A**), purification by solid phase extraction and elution of the RP cartridge, the radioligand was formulated manually in sterile isotonic saline containing 10% of ethanol (*v*/*v*) as described above. Analytical HPLC (system **B**) of the final product spiked with the non-radioactive reference compound **TA3** confirmed the identity of [^18^F]**TA3** (see Figure 5). The radioligand was obtained with a radiochemical purity higher than 99%, a radiochemical yield of 41.7% ± 6.3% (*n* = 3), and a specific activity of 142.6 ± 35.2 GBq/µmol (EOS; *n* = 3) in a total synthesis time of 75 min.

Compared to the manual procedure, there were no significant differences regarding the radiochemical yield of [^18^F]**TA3** achieved in the automated process. Besides the high reproducibility of the automated radiosyntheses, higher starting activities could be used (5–7 GBq *vs.* max. 3 GBq) and thus a significantly increased specific activity of [^18^F]**TA3** was obtained (143 GBq/µmol *vs.* 60 GBq/µmol).

### 2.3. In Vitro Autoradiographic Studies in Rat Brain

**Figure 6:**
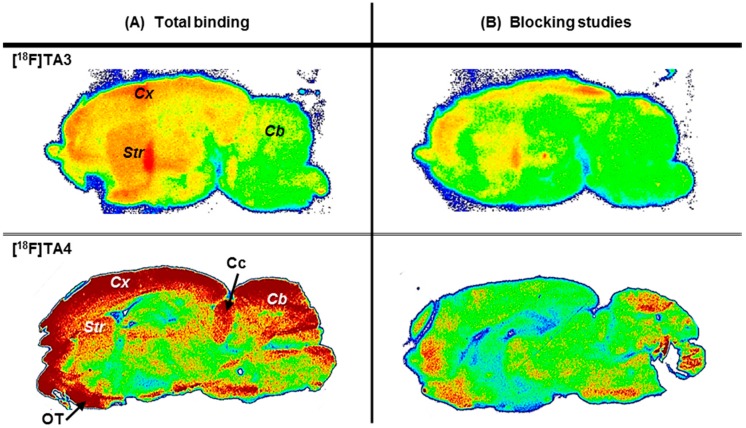
Representative autoradiographic images of sagittal rat brain slices: (**A**) *In vitro* distribution of activity after incubation with ~1 MBq/mL of [^18^F]**TA3** or [^18^F]**TA4**; (**B**) Non-specific binding of [^18^F]**TA3** or [^18^F]**TA4** determined in the presence of 1 µM of **TA1** as blocking compound. *Abbreviations*: Cb—cerebellum, Cc—colliculi, Cx—cortex, OT—olfactory tubercle, Str—striatum.

*In vitro* autoradiographic studies were accomplished by incubating sagittal sections of rat brain with [^18^F]**TA3** or [^18^F]**TA4**. Non-specific binding of each radioligand was assessed by co-incubation with an excess of lead compound **TA1**. The images shown in Figure 6 indicate region-specific accumulation of both radioligands [^18^F]**TA3** and [^18^F]**TA4**, which is consistent with the distribution pattern of PDE2A protein in rat brain [9,11]. Therefore, we assume that the novel radioligands might be appropriate for *in vitro* imaging of PDE2A. Notably, a specific PDE2A-radioligand (e.g., ^3^H-labelled) is not commercially available and *in vitro* autoradiographic images of the PDE2A distribution in brain have not been published yet.

The radioligand [^18^F]**TA3** shows higher binding densities in cortex and striatum than in cerebellum, while the radioligand [^18^F]**TA4** also binds to the olfactory tubercle, colliculi and partly to cerebellum.

### 2.4. In Vivo Metabolism of [^18^F]**TA3** and [^18^F]**TA4** in Mice

**Figure 7:**
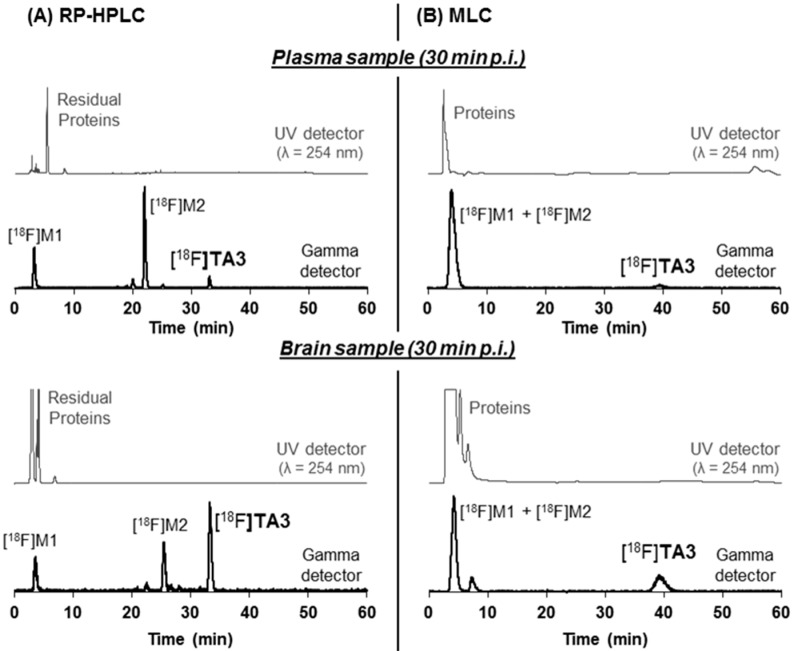
Representative *in vivo* metabolism study of mouse plasma and brain samples at 30 min p.i. of [^18^F]**TA3** (150 MBq): (**A**) RP-HPLC chromatograms of extracted samples (column: Reprosil-Pur C18-AQ, 250 × 4.6 mm, particle size: 5 µm; gradient: 10% → 90% → 10% MeCN/20 mM NH_4_OAc_aq._; flow: 1 mL/min); (**B**) MLC chromatograms of samples directly injected into the MLC system (column: Reprosil-Pur C18-AQ, 250 × 4.6 mm, particle size: 10 µm; gradient: 3% → 30% → 3% 1-PrOH/100 mM SDS_aq._, 10 mM Na_2_HPO_4aq._; flow: 1 mL/min).

*In vivo* metabolism of [^18^F]**TA3** and [^18^F]**TA4** was investigated in plasma and brain samples obtained from CD-1 mice at 30 min post injection of 150 MBq of each radioligand. Analysis of the samples was performed after protein precipitation and twofold extraction using an organic solvent. For both radioligands, a high fraction of radiometabolites was detected in plasma with only 8% and 6% of total activity representing non-metabolized [^18^F]**TA3** (Figure 7; recovery of total activity: 76%) and [^18^F]**TA4** (recovery: 83%), respectively. In brain samples, 50% of total activity were represented by intact [^18^F]**TA3** (Figure 7; recovery: 65%). No intact [^18^F]**TA4** was detectable in related brain samples (recovery: 85%).

The conventional extraction procedure is very work-intensive, time-consuming and—most important—an absolute quantification of the real composition in the samples is not possible due to 65%–85% recovery of total activity. In order to understand the impact of polar radiometabolites on the recovery yields, we used [^18^F]fluoride to investigate its extractability from denatured proteins *in vitro* taking into account that (i) defluorination is a common process in the *in vivo* metabolism of fluorine-bearing molecules; and (ii) [^18^F]fluoride is the most polar radiometabolite resulting from the metabolic degradation of ^18^F-compounds. Thus, pig plasma samples were incubated *in vitro* with [^18^F]fluoride and subsequent extraction was performed, applying the same protocol as for the *in vivo* metabolism studies, revealing that only 34% of the total activity could be recovered.

These findings indicate that the binding of polar radiometabolites, especially with an ionic character, to the denatured proteins is stronger than interactions of the proteins with the intact radioligand and thus, these radiometabolites are not completely extractable. Accordingly, the real percentage ratio of intact radioligand to its radiometabolites cannot be quantified by RP-HPLC based on samples obtained from an extraction procedure.

Therefore we used micellar HPLC (MLC), because this method allows the direct injection of plasma samples without deproteination. MLC was recently investigated by Nakao *et al.* [44] regarding their suitability for direct plasma metabolite analysis of PET radioligands. As eluent a mixture of an aqueous solution of sodium dodecyl sulphate (SDS), phosphate buffer and 1-propanol was used. According to the published procedure [44], a gradient mode was applied starting under micellar conditions to completely elute the protein fraction and release the protein bound ^18^F-compounds by the SDS micelles. Subsequent increase of the amount of 1-propanol as organic modifier leads to high submicellar conditions [44,45] resulting in the elution of the intact radioligand and its radio-metabolites.

Analysis of the MLC chromatograms revealed that only 5% of total activity in plasma are represented by intact radioligand [^18^F]**TA3** (Figure 7) or [^18^F]**TA4**. For the first time, the micellar HPLC method was also applied to evaluate mouse brain radiometabolites. In the analyzed brain samples, 29% and 4% of total activity were represented by intact [^18^F]**TA3** (Figure 7) and [^18^F]**TA4**, respectively.

The radio-chromatograms of the RP-HPLC (conventional extraction) and the micellar HPLC method displayed slightly different elution profiles (for [^18^F]**TA3** see Figure 7) which are probably caused by the various retention mechanisms in these two systems. RP-HPLC revealed the presence of two major radiometabolites ([^18^F]M1 and [^18^F]M2) in plasma and brain for each radioligand. It is supposed that the second radiometabolite [^18^F]M2 elutes within the first fraction under micellar conditions.

Furthermore, comparing the results of the RP-HPLC (for [^18^F]**TA3**: *t_R_* = 33 min, see Figure 7) and the MLC (for [^18^F]**TA3**: *t_R_* = 39 min, see Figure 7), slightly different concentrations of the detected radioligands in the mouse plasma samples (e.g., for [^18^F]**TA3**: RP: 8% *vs.* MLC: 5%) and brain homogenates (e.g., for [^18^F]**TA3**: RP: 50% *vs.* MLC: 29%) can be observed. These findings might be caused by insufficient extraction of radioactive compounds (recovery: 65%–85%) from the precipitated proteins, mainly of the more polar radiometabolites detected by RP-HPLC (for [^18^F]**TA3**: [^18^F]M1: *t_R_* = 3 min; [^18^F]M2: *t_R_* = 22 min, see Figure 7). Therefore we conclude that only MLC provides the detection of 100% of the real composition in the analyzed samples due to direct injection into the micellar HPLC system.

In comparison with the intact radioligands, the shorter retention times of [^18^F]M1 and [^18^F]M2 observed in the radio-chromatograms of the RP-HPLC (for [^18^F]**TA3** see Figure 7) point to a higher polarity of these radiometabolites. The similar elution profiles of extracted plasma and brain samples (for [^18^F]**TA3** see Figure 7) indicate that both radiometabolites may cross the blood-brain barrier. The significant accumulation of the highly polar radiometabolite [^18^F]M1 in brain could be explained by cytochrome P450 enzyme induced metabolic degradation of the ^18^F-bearing alkyl side chains in [^18^F]**TA3** or [^18^F]**TA4** and formation of the corresponding brain penetrating ^18^F-alkyl alcohols, aldehydes or carboxylic acids [46,47]. Regarding the formation of the radiometabolite [^18^F]M2, the related mechanism of metabolic degradation and thus the molecular structure of [^18^F]M2 remain unclear.

### 2.5. PET/MR Studies of [^18^F]**TA3** in Mice

The metabolism studies indicated that the metabolic stability of [^18^F]**TA3** in mouse is significantly higher than that of [^18^F]**TA4**. Thus, further *in vivo* studies using dynamic PET imaging were performed only with [^18^F]**TA3**.

**Figure 8:**
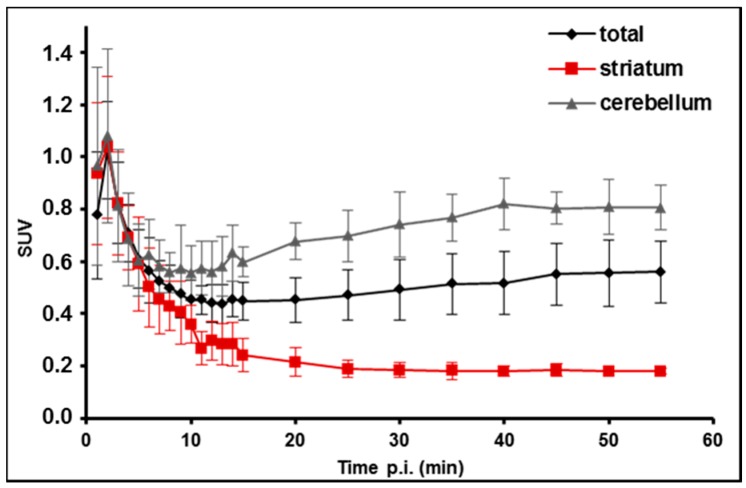
Averaged time-activity curves of [^18^F]**TA3** in CD-1 mice (*n* = 4) with standard uptake values (SUV) in whole brain (total), striatum, and cerebellum.

For PET/MR baseline studies of [^18^F]**TA3** in anaesthetized CD-1 mice, the radioligand was injected intravenously (9.7 ± 1.3 MBq, SA_EOS_ ~70 GBq/µmol, *n* = 4), and whole body scans were performed for 60 min in listmode with a Mediso nanoScan PET/MR scanner followed by dynamic reconstruction. Time-activity curves (TACs) were generated for regions of interest such as whole brain, striatum, and cerebellum (Figure 8).

As presented in Figure 8, the TACs between 0 and 55 min p.i. show a fast wash out of activity from the striatum while a constantly increased uptake in the cerebellum was observed. This finding is not consistent with the distribution of PDE2A protein in murine brain which corresponds the *in vitro* autoradiography of [^18^F]**TA3** in rat brain slices (see Figure 6), but likely reflects the accumulation of the radiometabolite [^18^F]M2 capable of penetrating the blood-brain barrier as detected by RP-HPLC analysis of extracted brain samples (see Figure 7). With regard to that, we assume that the constantly increasing uptake of activity in the cerebellum as a non-target region of a PDE2A radioligand, which is in contrast to the fast wash out of activity from the target region striatum, indicates specific binding of the structurally not analyzed radiometabolite [^18^F]M2 of [^18^F]**TA3** to an unknown target in this brain region.

## 3. Experimental Section

### 3.1. General Information

Chemicals were purchased from standard commercial sources in analytical grade and were used without further purification. Radio-/TLCs were performed on pre-coated silica gel plates (Alugram^®^ Xtra SIL G/UV_254_; Polygram^®^ SIL G/UV_254_, Roth, Karlsruhe, Germany). The compounds were localized at 254 nm (UV lamp) and/or by staining with aqueous KMnO_4_ solution or ninhydrin solution. Radio-TLC was recorded using a bioimaging analyzer system (BAS-1800 II, Fuji Photo Film, Co. Ltd., Tokyo, Japan) and images were evaluated with Aida 2.31 software (raytest Isotopenmessgeräte GmbH, Straubenhardt, Germany). Column chromatography was conducted on silica gel (0.06–0.20 mm, Roth). HPLC separations were performed on JASCO systems equipped with UV detectors from JASCO and activity detectors from raytest Isotopenmessgeräte GmbH (GABI Star, Straubenhardt, Germany).

HPLC columns and conditions were: System **A**, semi-preparative HPLC (column: Reprosil-Pur C18-AQ, 250 × 10 mm, particle size: 10 µm; eluent: 50% MeCN/20 mM NH_4_OAc_aq._; flow: 3 mL/min for [^18^F]**TA3** or 3.5 mL/min for [^18^F]**TA4**; ambient temperature; UV detection at 254 nm); system **B**, analytical HPLC (column: Reprosil-Pur C18-AQ, 250 × 4.6 mm, particle size: 5 µm; gradient: 0–10 min: 10% MeCN, 10–35 min: 10% → 90% MeCN, 35–45 min: 90% MeCN, 45–50 min: 90% → 10% MeCN, 50–60 min: 10% MeCN/20 mM NH_4_OAc_aq._; isocratic: 44% MeCN/20 mM NH_4_OAc_aq._; flow: 1 mL/min; ambient temperature; UV detection at 254 nm). The NH_4_OAc concentration stated as 20 mM NH_4_OAc_aq._ corresponds to the concentration in the aqueous component of an eluent mixture.

Specific activity was determined on the base of a calibration curve carried out under isocratic HPLC conditions (44% MeCN/20 mM NH_4_OAc_aq._; system **B**) using chromatograms obtained at 270 nm as an appropriate maximum of UV absorbance.

NMR spectra (^1^H, ^13^C, ^19^F) were recorded on Mercury 300/Mercury 400 (Varian, Palo Alto, CA, USA) or Fourier 300/Avance DRX 400 Bruker (Billerica, MA, USA) instruments. The hydrogenated residue of deuteriated solvents and/or tetramethylsilane (TMS) were used as internal standards for ^1^H-NMR (CDCl_3_, δ = 7.26; DMSO-*d*_6_, δ = 2.50) and ^13^C-NMR (CDCl_3_, δ = 77.2; DMSO-*d*_6_, δ = 39.5). The chemical shifts (δ) are reported in ppm (s, singlet; d, doublet; t, triplet; qui, quintet; m, multiplet) and the corresponding coupling constants (*J*) are reported in Hz. High resolution mass spectra (ESI +/−) were recorded on an Esquire 300Plus instrument (Bruker; equipped with ion trap). No-carrier-added (n.c.a.) [^18^F]fluoride (*t*_1/2_ = 109.8 min) was produced via the [18O(p,n)18F] nuclear reaction by irradiation of [18O]H_2_O (Hyox 18 enriched water, Rotem Industries Ltd, Beer-Sheba, Israel) on a Cyclone^®^18/9 (iba RadioPharma Solutions, Louvain-la-Neuve, Belgium) with fixed energy proton beam using Nirta^®^ [^18^F]fluoride XL target.

### 3.2. Syntheses

The synthesis of the lead compound **TA1** is already reported [38] and involves five steps, which were partly optimized. Furthermore, the patent does not provide NMR data of the published compounds **1**–**4** or **TA1**, which are reported herein.

*6-Methoxy-2-(4-methyl-1H-imidazol-1-yl)-3-nitropyridine* (**1**) [38]. To 4.02 g (1.6 eq.) 4-methylimidazole dissolved in CHCl_3_ (8 mL, instead of DMF [38]) a solution of 4-(dimethylamino) pyridine (DMAP, 0.37 g, 10 mol %) in CHCl_3_ (2 mL) was added dropwise. The reaction mixture was stirred at 0 °C for 10 min followed by the addition of triethylamine (TEA, 12.73 mL, 3 eq.; instead of solid KOH [38]) and stirring at 0 °C for further 10 min. To this reaction mixture was added dropwise 2-chloro-6-methoxy-3-nitropyridine (5.77 g, 1 eq.) in CHCl_3_ (14 mL). After 30 min at 0 °C the mixture was stirred at ambient temperature overnight. The mixture was washed twice with water and aq. NaCl saturated solution (20 mL). The aqueous phase was extracted with CHCl_3_ (20 mL). The organic phase was dried over Na_2_SO_4_ and filtered. Evaporation of the solvent and subsequent purification by column chromatography (EtOAc/*n*-hexane, 1:2, *v*/*v*) afforded **1** as a yellow solid (6.65 g, 93%). ^1^H-NMR (400 MHz, CDCl_3_): δ (ppm) = 2.27 (s, 3H); 4.03 (s, 3H); 4.75 (s, 2H); 6.78 (d, ^3^*J* = 8.8, 1H); 6.90 (dd, ^4^*J* = 2.0, ^4^*J* = 1.2, 1H); 7.94 (d, ^4^*J* = 1.2, 1H); 8.23 (d, ^3^*J* = 8.8, 1H).

*6-Methoxy-2-(4-methyl-1H-imidazol-1-yl)pyridin-3-amine* (**2**) [38]. Compound **1** (6.62 g, 1 eq.) was dissolved in absolute EtOH (180 mL) and after addition of palladium on charcoal (0.20 g, 10%) in absolute EtOH (20 mL) the reaction mixture was hydrogenated under pressure (1.5–2 bar instead of 10 to 15 bar [38]) at ambient temperature (instead of 40 °C [38]). Afterwards the mixture was stirred overnight and filtered over kieselguhr (suspended in EtOH). The red brown filtrate was concentrated under reduced pressure and the residue was treated with cold methyl *tert*-butyl ether (MTBE, 20 mL). The mixture was stirred at 0 °C for 30 min and stored at 4 °C (fridge) overnight to precipitate the product. The light brown solid was filtered and dried in a desiccator under vacuum for two days. This procedure was repeated twice with the filtrate and the dry precipitates were combined to afford 4.54 g (79%) of **2**. ^1^H-NMR (300 MHz, DMSO-*d*_6_): δ (ppm) = 2.17 (s, 3H); 3.74 (s, 3H); 4.75 (s, 2H); 6.67 (d, ^3^*J* = 8.4, 1H); 7.29 (m, 1H); 7.35 (d, ^3^*J* = 8.4, 1H); 7.96 (d, ^4^*J* = 1.2, 1H).

*2-Methoxy-7-methylimidazo[5,1-c]pyrido[2,3-e][1,2,4]triazine* (**3**) [38]. Under ice bath cooling, compound **2** (100 mg, 1 eq.) was dissolved in CH_3_COOH (30 mL) followed by addition of aq. NaNO_2_ solution (51 mg, 1.5 eq. in 500 µL water) and water (2 mL). Precipitation of a yellow solid started immediately. The reaction mixture was stirred for 30 min (instead of 1–2 h [38]) and the precipitate was filtered, washed with ethyl acetate and water, and dried in a desiccator under vacuum for three days (instead of re-crystallization from *iso*-propanol or column chromatography [38]). 101 mg (96%) of **3** as a yellow solid were obtained. ^1^H-NMR (400 MHz, DMSO-*d*_6_): δ (ppm) = 2.77 (s, 3H); 4.13 (s, 3H); 7.21 (d, ^3^*J* = 8.8, 1H); 8.72 (d, ^3^*J* = 8.8, 1H); 8.97 (s, 1H).

*9-Bromo-2-methoxy-7-methylimidazo[5,1-c]pyrido[2,3-e][1,2,4]triazine* (**4**) [38]. A solution of compound **3** (0.74 g, 1 eq.; instead of 4-methoxy-7-methyl-imidazo[5,1-c]pyrido[2,3-e][1,2,4]triazine [38]) in CH_2_Cl_2_ (50 mL; instead of MeCN [38]) was stirred for 10 min under ice bath cooling. A suspension of *N*-bromosuccinimide (NBS, 0.92 g, 1.5 eq.) in CH_2_Cl_2_ (10 mL) was added dropwise and the round bottom flask was covered using aluminum foil. The reaction was stirred in an ice bath for 5 h and overnight at ambient temperature (instead of stirring at ambient temperature for 16 h [38]). The mixture was washed once with aq. saturated solutions of Na_2_SO_3_, NaHCO_3_, and NaCl and water (20 mL each). The aqueous phase was extracted with CH_2_Cl_2_ (20 mL). The organic phase was dried over Na_2_SO_4_ and filtered. Evaporation of the solvent and subsequent purification by column chromatography (EtOAc/CH_2_Cl_2_, 1:6 to 1:4, *v*/*v*) afforded a yellow solid of **4** (0.74 g, 73%). ^1^H-NMR (300 MHz, CDCl_3_): δ (ppm) = 2.85 (s, 3H); 4.19 (s, 3H); 7.04 (d, ^3^*J* = 8.8, 1H); 8.57 (d, ^3^*J* = 8.8, 1H).

*9-(5-Butoxy-2-fluorophenyl)-2-methoxy-7-methylimidazo[5,1-c]pyrido[2,3-e][1,2,4]triazine* (**TA1**) [38]. The synthesis of the lead compound **TA1** was performed as described in the General Suzuki Coupling Procedure [38]. Subsequent evaporation of the solvent at 60 °C and purification by column chromatography (EtOAc/CH_2_Cl_2_, 1:6, *v*/*v*) afforded a yellow solid of **TA1** (0.34 g, 81%). ^1^H-NMR (300 MHz, DMSO-*d*_6_): δ (ppm) = 0.90 (t, ^3^*J* = 7.4, 3H); 1.35–1.50 (m, 2H); 1.60–1.75 (m, 2H); 2.79 (s, 3H); 3.64 (s, 3H); 3.98 (t, ^3^*J* = 6.4, 2H); 7.07–7.14 (m, 1H), 7.10 (d, ^3^*J* = 8.7, 1H); 7.21 (dd, ^4^*J* = 5.7, ^5^*J* = 3.1, 1H); 7.29 (t, ^3^*J* = 9.2, 1H); 8.64 (d, ^3^*J* = 8.7, 1H). ^13^C-NMR (75 MHz, DMSO-*d*_6_): δ (ppm) = 12.3 (s); 13.6 (s); 18.7 (s); 30.7 (s); 54.2 (s); 68.0 (s); 112.1 (s); 115.9 (d, ^2^*J* = 22.5); 117.3 (d, ^3^*J* = 8.3); 117.5 (d, ^3^*J* = 1.5); 120.2 (d, ^2^*J* = 16.5); 127.8 (s); 131.6 (s); 133.5 (s); 136.6 (s); 138.9 (s); 140.6 (s); 154.2 (d, ^4^*J* = 2.3); 154.8 (d, ^1^*J* = 238.5); 163.8 (s). ^19^F-NMR (282 MHz, DMSO-*d*_6_): δ (ppm) = −121.7 (ddd, ^1^*J* = 9.2, ^2^*J* = 5.7, ^2^*J* = 4.3). HR-MS (ESI): 382.17 [M+H]^+^(Lit.: 382 [38]).

*4-Fluoro-3-(2-methoxy-7-methylimidazo[5,1-c]pyrido[2,3-e][1,2,4]triazin-9-yl)phenol* (**TA1a**) A solution of compound **TA1** (1.00 g, 1 eq.) in dry CH_2_Cl_2_ (50 mL) was placed in a 100 mL twin-neck flask under ice bath cooling and argon atmosphere. Afterwards boron tribromide (8 mL, 3.05 eq.) in CH_2_Cl_2_ (1 M solution) was added dropwise over a period of 45 min whereby the color of the mixture changed from yellow to dark red. The reaction mixture was stirred at 0 °C (ice bath) for 1 h and then warmed up to ambient temperature. The mixture was quenched by adding it dropwise to ice water (100 mL) and the organic phase was washed twice with water (10 mL each). The aqueous phase was extracted twice with CH_2_Cl_2_ (5 mL each). The combined organic phases were dried over Na_2_SO_4_ and filtered. Evaporation of the solvent and subsequent purification by column chromatography (EtOAc/CH_2_Cl_2_, 1:6 to 100% EtOAc, *v*/*v*) afforded a yellow solid of **TA1a** (0.84 g, 98%). ^1^H-NMR (300 MHz, DMSO-*d*_6_): δ (ppm) = 2.82 (s, 3H); 3.52 (s, 3H); 6.92 (ddd, ^3^*J* = 9.0, ^4^*J* = 4.2, ^4^*J* = 3.1, 1H); 7.04 (dd, ^4^*J* = 5.7, ^4^*J* = 3.1, 1H); 7.16 (d, ^3^*J* = 9.0, 1H); 7.19 (t, ^3^*J* = 9.0, 1H); 8.71 (d, ^3^*J* = 9.0, 1H); 9.65 (s, 1H).

#### 3.2.1. General Procedure for the Preparation of Fluoroalkylated Triazine Derivatives **TA2**–**4**

To a solution of compound **TA1a** (1 eq.) in MeCN (10 mL), K_2_CO_3_ (3 eq.) and the fluoroalkylating agent (1.5 eq.) were added. The yellow suspension was stirred at 70–80 °C for 5 h and at ambient temperature overnight. The mixture was filtered and the solvent was evaporated. The residue was dissolved in CH_2_Cl_2_ (10 mL), washed with water (5 mL) and then with citric acid (5 mL, 25%). The aqueous phase was extracted with CH_2_Cl_2_ (2 mL). The combined organic phases were dried over Na_2_SO_4_, filtered and evaporation of the solvent yielded the crude fluoroalkylated triazine derivative.

*9-(2-Fluoro-5-(2-fluoroethoxy)phenyl)-2-methoxy-7-methylimidazo[5,1-c]pyrido[2,3-e] [1,2,4]triazine* (**TA2**) The fluoroalkylation procedure described above was used with 100 mg of **TA1a** and 38 µL of 1-fluoro-2-iodoethane and afforded after column chromatography (EtOAc/CH_2_Cl_2_, 1:4 to 1:1, *v*/*v*) a yellow solid of **TA2** (60 mg, 54%). ^1^H-NMR (300 MHz, DMSO-*d*_6_): δ (ppm) = 2.82 (s, 3H); 3.48 (s, 3H); 4.23 (t, ^2^*J* = 7.5, ^3^*J* = 30.3, ^3^*J* = 3.9, 1H); 4.33 (t, ^2^*J* = 7.8, ^3^*J* = 30.3, ^3^*J* = 3.9, 1H) 4.66 (t, ^2^*J* = 7.8, ^2^*J* = 48.0, ^3^*J* = 3.9, 1H), 4.82 (t, ^2^*J* = 7.5, ^2^*J* = 48.0, ^3^*J* = 3.9, 1H); 7.15 (d, ^3^*J* = 9.0, 1H); 7.18–7.22 (m, 1H); 7.26–7.31 (m, 1H); 7.35 (t, ^3^*J* = 9.3, 1H); 8.69 (d, ^3^*J* = 9.0, 1H). ^13^C-NMR (75 MHz, DMSO-*d*_6_): δ (ppm) = 12.4 (s); 54.2 (s); 54.2 (s); 67.9 (d, ^3^*J* = 18.8); 82.1 (d, ^1^*J* = 165.8); 112.2 (s); 116.1 (d, ^2^*J* = 23.0); 117.57 (s); 117.7 (d, ^3^*J* = 2.0); 120.4 (d, ^2^*J* = 16.4); 127.9 (s); 131.4 (s); 133.6 (s); 136.6 (s); 139.0 (s); 140.7 (s); 153.7 (d, ^4^*J* = 1.9); 155.1 (d, ^1^*J* = 239.3); 163.9 (s). ^19^F-NMR (282 MHz, DMSO-*d*_6_): δ (ppm) = −121.3 to −121.5 (m); -222.6 (tt, ^2^*J* = 48.0, ^3^*J* = 30.3). HR-MS (ESI): 372.13 [M+H]^+^.

*9-(2-Fluoro-5-(2-fluoropropoxy)phenyl)-2-methoxy-7-methylimidazo[5,1-c]pyrido[2,3-e][1,2,4]-triazine* (**TA3**) The fluoroalkylation procedure described above was performed with 140 mg of **TA1a** and 66 µL of 1-fluoro-3-iodopropane and afforded after column chromatography (EtOAc/CH_2_Cl_2_, 1:4, *v*/*v*) a yellow solid of **TA3** (120 mg, 75%). ^1^H-NMR (400 MHz, DMSO-*d*_6_): δ (ppm) = 2.10 (dqui, ^2^*J* = 12.2, ^3^*J* = 25.6, ^3^*J* = 6.0, 2H); 2.81 (s, 3H); 3.47 (s, 3H); 4.10 (t, ^2^*J* = 12.4, ^3^*J* = 6.0, 2H); 4.55 (t, ^2^*J* = 12.0, ^2^*J* = 47.2, ^3^*J* = 6.0, 1H), 4.67 (t, ^2^*J* = 11.6, ^2^*J* = 47.2, ^3^*J* = 6.0, 1H); 7.13 (d, ^3^*J* = 8.8, 1H); 7.13–7.18 (m, 1H);7.26 (dd, ^4^*J* = 5.7, ^5^*J* = 3.2, 1H); 7.32 (t, ^3^*J* = 9.2, 1H); 8.67 (d, ^3^*J* = 8.8, 1H). ^13^C-NMR (101 MHz, DMSO-*d*_6_): δ (ppm) = 12.4 (s); 29.7 (d, ^2^*J* = 19.7); 54.2 (s); 64.4 (s); 80.8 (d, ^1^*J* = 162.3); 112.1 (s); 116.0 (d, ^2^*J* = 23.1); 117.4 (d, ^3^*J* = 8.1); 117.7 (d, ^3^*J* = 1.9); 120.3 (d, ^2^*J* = 16.5); 127.8 (s); 131.5 (s); 133.6 (s); 136.6 (s); 138.9 (s); 140.6 (s); 154.0 (d, ^4^*J* = 2.0); 155.0 (d, ^1^*J* = 241.3); 163.8 (s). ^19^F-NMR (376 MHz, DMSO-*d*_6_): δ (ppm) = −121.5 to −121.6 (m); -220.9 (tt, ^2^*J* = 47.2, ^3^*J* = 25.6). HR-MS (ESI): 386.14 [M+H]^+^.

*9-(2-Fluoro-5-(2-fluorobutoxy)phenyl)-2-methoxy-7-methylimidazo[5,1-c]pyrido[2,3-e] [1,2,4]triazine* (**TA4**) The fluoroalkylation procedure described above was performed with 100 mg of **TA1a** and 50 µL of 1-bromo-4-fluorobutane and afforded after column chromatography (EtOAc/CH_2_Cl_2_, 1:4, *v*/*v*) a yellow solid of **TA4** (120 mg, 99%). ^1^H-NMR (400 MHz, DMSO-*d*_6_): δ (ppm) = 1.70–1.86 (m, 4H); 2.80 (s, 3H); 3.46 (s, 3H); 4.01 (t, ^2^*J* = 10.4, ^3^*J* = 6.0, 2H); 4.42 (t, ^2^*J* = 11.6, ^2^*J* = 47.6, ^3^*J* = 6.0, 1H), 4.54 (t, ^2^*J* = 11.6, ^2^*J* = 47.6, ^3^*J* = 5.6, 1H); 7.08–7.16 (m, 1H); 7.11 (d, ^3^*J* = 8.8, 1H); 7.22 (dd, ^4^*J* = 5.6, ^5^*J* = 3.2, 1H); 7.29 (t, ^3^*J* = 9.2, 1H); 8.66 (d, ^3^*J* = 8.8, 1H).

#### 3.2.2. General Procedure for the Preparation of alkyl bis(4-methylbenzenesulfonates)

The synthesis of various alkyl bis(4-methylbenzenesulfonates) is already reported [48] and was performed according to the literature. A solution of propane-1,3-diol or butane-1,4-diol (1 eq.) in CH_2_Cl_2_ (5 mL) was stirred at 0 °C (ice bath) for 10 min. The round bottom flask was covered using aluminium foil and 4 eq. of triethylamine (TEA) were added dropwise. Afterwards a solution of 4-methylbenzenesulfonyl chloride (2 eq.) in CH_2_Cl_2_ (10 mL) was added over a period of 15–30 min followed by the addition of 10 mol % of DMAP. The reaction mixture was stirred each for 1 h at 0 °C and at ambient temperature. The white precipitate (triethylamine hydrochloride) was filtered off. The light yellow filtrate was washed once with each 1 M HCl, aq. saturated solution of NaHCO_3_, water, and aq. saturated solution of NaCl. The aqueous phase was extracted once with CH_2_Cl_2_. The organic phase was dried over Na_2_SO_4_ and filtered. Evaporation of the solvent and subsequent purification by column chromatography (EtOAc/*n*-hexanes, 1:3 to 1:1, *v*/*v*) afforded a white solid (≥60% of each: propane-1,3-diyl bis(4-methylbenzenesulfonate) and butane-1,4-diyl bis(4-methyl-benzenesulfonate)), identified by ^1^H-NMR.

#### 3.2.3. General Procedure for the Preparation of Tosylate Precursors **TA3a** and **TA4a**

To a solution of compound **TA1a** (1 eq.) in MeCN (10 mL), K_2_CO_3_ (4 eq.) and the alkyl bis(4-methylbenzenesulfonate) (2 eq.) were added. The yellow suspension was stirred at 60–70 °C for 5 h and at ambient temperature overnight. In the case of an incomplete reaction (TLC) the mixture was heated to 60–70 °C for 5 h again and stirred at ambient temperature overnight. The mixture was filtered and the solvent was evaporated. The residue was resolved in CH_2_Cl_2_ (10 mL), washed with water (5 mL) and then with citric acid (5 mL, 25%). The aqueous phase was extracted with CH_2_Cl_2_ (2 mL). The combined organic phases were dried over Na_2_SO_4_, filtered and evaporation of the solvent yielded the crude tosylate precursors.

*3-(4-Fluoro-3-(2-methoxy-7-methylimidazo[5,1-c]pyrido[2,3-e][1,2,4]triazine-9-yl)phenoxy)propyl 4-methylbenzenesulfonate* (**TA3a**) The procedure for tosylate precursors described above was performed with 170 mg of **TA1a** and 410 mg of propane-1,3-diyl bis(4-methylbenzenesulfonate), and afforded after column chromatography (EtOAc/CH_2_Cl_2_, 1:6 to 1:4, *v*/*v*) a yellow solid of **TA3a** (170 mg, 60%). ^1^H-NMR (400 MHz, DMSO-*d*_6_): δ (ppm) = 2.03 (qui, ^2^*J* = 24.4, ^3^*J* = 6.0, 2H); 2.34 (s, 3H); 2.83 (s, 3H); 3.46 (s, 3H); 3.97 (t, ^2^*J* = 12.0, ^3^*J* = 6.0, 2H); 4.19 (t, ^2^*J* = 12.0, ^3^*J* = 6.0, 1H); 7.02–7.07 (m, 1H); 7.13–7.17 (m, 1H); 7.16 (d, ^3^*J* = 8.8, 1H); 7.31 (t, ^3^*J* = 9.2, 1H); 7.41 (d, ^3^*J* = 8.4, 2H); 7.76 (dd, ^3^*J* = 8.4, ^4^*J* = 1.6, 2H); 8.71 (d, ^3^*J* = 8.8, 1H). ^13^C-NMR (101 MHz, DMSO-*d*_6_): δ (ppm) = 12.4 (s); 21.0 (s); 28.1 (s); 54.2 (s); 64.1 (s); 67.6 (s); 112.2 (s); 116.0 (d, ^2^*J* = 23.3); 117.3 (d, ^3^*J* = 8.3); 117.6 (d, ^3^*J* = 1.7); 120.3 (d, ^2^*J* = 16.4); 127.5 (s); 127.9 (s); 130.1 (s); 131.5 (s); 132.2 (s); 133.6 (s); 136.6 (s); 139.0 (s); 140.7 (s); 153.8 (d, ^4^*J* = 2.0); 155.0 (d, ^1^*J* = 241.4); 163.9 (s). ^19^F-NMR (376 MHz, DMSO-*d*_6_): δ (ppm) = −121.5 to −121.6 (m). HR-MS (ESI): 538.16 [M+H]^+^.

*4-(4-Fluoro-3-(2-methoxy-7-methylimidazo[5,1-c]pyrido[2,3-e][1,2,4]triazine-9-yl)phenoxy)butyl 4-methylbenzenesulfonate* (**TA4a**) The procedure for tosylate precursors described above was performed with 200 mg of **TA1a** and 490 mg of butane-1,4-diyl bis(4-methylbenzenesulfonate), and afforded after column chromatography (EtOAc/CH_2_Cl_2_, 1:6 to 1:4, *v*/*v*) an orange to red solid of **TA4a** (220 mg, 65%). ^1^H-NMR (300 MHz, DMSO-*d*_6_): δ (ppm) = 1.62–1.80 (m, 4H); 2.38 (s, 3H); 2.82 (s, 3H); 3.46 (s, 3H); 3.93 (t, ^2^*J* = 11.7, ^3^*J* = 5.8, 2H); 4.09 (t, ^2^*J* = 11.7, ^3^*J* = 5.9, 2H); 7.04–7.11 (m, 1H); 7.15 (d, ^3^*J* = 9.0, 1H); 7.19 (dd, ^4^*J* = 5.7, ^5^*J* = 3.1, 1H); 7.31 (t, ^3^*J* = 9.2, 1H); 7.45 (dd, ^3^*J* = 8.4, ^4^*J* = 0.6, 2H); 7.78 (dd, ^3^*J* = 8.4, ^4^*J* = 0.6, 2H); 8.69 (d, ^3^*J* = 9.0, 1H). ^13^C-NMR (75 MHz, DMSO-*d*_6_): δ (ppm) = 13.0 (s); 21.72 (s); 25.3 (s); 25.8 (s); 54.9 (s); 68.2 (s); 71.3 (s); 112.9 (s); 116.7 (d, ^2^*J* = 23.0); 118.0–118.4 (m); 121.0 (d, ^2^*J* = 16.4); 128.2 (s); 128.6 (s); 130.8 (s); 132.2 (s); 133.2 (s); 134.3 (s); 137.3 (s); 139.7 (s); 141.4 (s); 145.5 (s); 155.6 (d, ^1^*J* = 238.6); 154.7 (d, ^4^*J* = 1.9); 164.6 (s). ^19^F-NMR (282 MHz, DMSO-*d*_6_): δ (ppm) = −121.7 (ddd, ^1^*J* = 9.2, ^2^*J* = 5.7, ^2^*J* = 4.3). HR-MS (ESI): 574.15 [M+Na]^+^.

### 3.3. In Vitro PDE2A Affinity Assay

The inhibitory potencies of **TA1**–**4** for human recombinant PDE2A and PDE10A proteins were determined by BioCrea GmbH (Radebeul, Germany) [38].

### 3.4. Radiochemistry

#### 3.4.1. Manual Radiosyntheses of [^18^F]**TA3** and [^18^F]**TA4**

No-carrier-added [^18^F]fluoride was trapped on a Chromafix^®^ 30 PS-HCO_3_^−^ cartridge (MACHEREY-NAGEL GmbH & Co. KG, Düren, Germany). The activity was eluted with 300 µL of an aqueous solution of K_2_CO_3_ (1.78 mg, 12.9 µmol) into a 4 mL V-vial and Kryptofix 2.2.2 (K_2.2.2_, 11.2 mg, 29.7 µmol) in 1 mL MeCN was added. The aqueous [^18^F]fluoride was azeotropically dried under vacuum and nitrogen flow within 7–10 min using a Discover PETwave Microwave CEM^®^ (75 W, 50–60 °C, power cycling mode). Two aliquots of MeCN (2 × 1.0 mL) were added during the drying procedure and the final complex was dissolved in 500 µL MeCN ready for radiolabelling. The reactivity of the anhydrous K^+^/[^18^F]F^−^/K_2.2.2_-carbonate complex as well as the reproducibility of the drying procedure were checked via the standard reaction with 2 mg (5.4 µmol) of ethylene glycol ditosylate (Sigma-Aldrich, Munich, Germany) at 80 °C for 10 min in MeCN.

Optimization of the aliphatic radiolabelling procedures of the tosylates **TA3a** or **TA4a** was performed by varying the amount of precursor (1–3 mg in 500 µL MeCN) and reaction time (up to 20 min) under conventional heating at 80 °C. After 5, 10, 15 and 20 min, aliquots of the reaction mixtures were analyzed by radio-TLC (EtOAc/DCM, 1:1, *v*/*v*). The crude reaction mixtures of [^18^F]**TA3** or [^18^F]**TA4** were diluted with water (1:1, *v*/*v*) and applied to an isocratic semi-preparative HPLC (system **A**) for isolation of the desired radioligands ([^18^F]**TA3**: *t_R_* = 27–29 min; [^18^F]**TA4**:*t_R_* = 31–33 min). The collected fractions were analyzed by radio-TLC, diluted with water, passed through a Sep-Pak^®^ C18 Plus light cartridge (Waters, Milford, MA, USA; pre-conditioned with 20 mL of absolute EtOH and 60 mL water), and eluted with 0.75 mL of absolute EtOH. For biological investigations, the solvent was evaporated at 70 °C under a gentle nitrogen stream. Then, the radioligands were formulated in sterile isotonic saline containing 10% EtOH (*v*/*v*).

The identities of each radioligand were verified by analytical radio-HPLC (system **B**) of samples of [^18^F]**TA3** or [^18^F]**TA4** spiked with the corresponding non-radioactive reference compounds using a gradient and an isocratic method.

#### 3.4.2. Automated Radiosynthesis of [^18^F]**TA3**

**Scheme 3:**
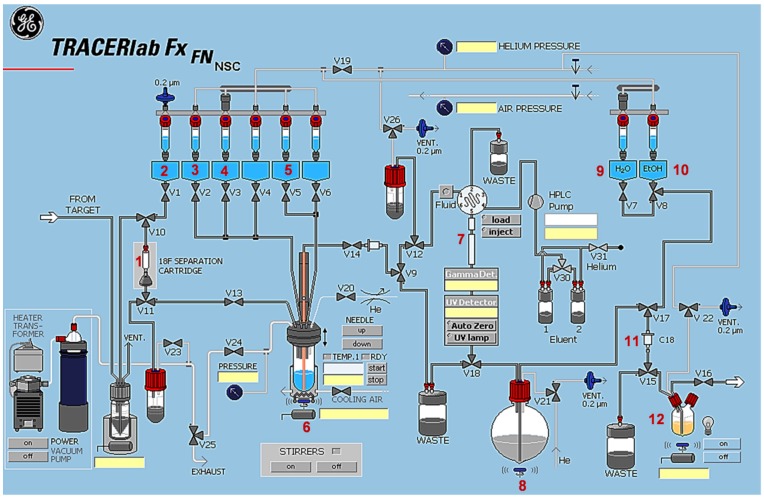
Tracer Lab™ FX-FN synthesis module for the automated radiosynthesis of [^18^F]**TA3**: (**1**) Chromafix^®^ 30 PS-HCO_3_^−^ cartridge; (**2**) K_2_CO_3_ (1.78 mg in 0.4 mL H_2_O); (**3**) K_2.2.2_ (11.2 mg in 1 mL MeCN); (**4**) 1 mg **TA3a** in 1 mL MeCN; (**5**) 2 mL H_2_O; (**6**) reaction vessel; (**7**) Reprosil-Pur C18-AQ (250 × 10 mm; particle size: 10 µm; eluent: 50% MeCN/20 mM NH_4_OAc_aq._; flow: 3 mL/min; ambient temperature; UV detection at 254 nm); (**8**) 40 mL H_2_O; (**9**) 2 mL H_2_O; (**10**) 1 mL absolute EtOH; (**11**) Sep Pak^®^ C18 light cartridge; (**12**) product vial.

Automated radiosyntheses were performed in a TRACERlab™ FX F-N synthesis module (GE Healthcare, Waukesha, WI, USA) equipped with a PU-980 pump (JASCO, Gross-Umstadt, Germany), a WellChrom K-2001 UV detector (KNAUER GmbH, Berlin, Germany), a NaI(Tl)-counter and automated data acquisition (NINA software version 4.8 rev. 4, Nuclear Interface GmbH, Dortmund, Germany). The conditions for the optimized manual ^18^F-labelling of the tosylate precursor **TA3a** as well as for the isolation and purification of the radioligand were used for the automated radiosynthesis of [^18^F]**TA3** and set up as depicted in Scheme 3. No-carrier-added [^18^F]fluoride was trapped on a Chromafix^®^ 30 PS-HCO_3_^−^ cartridge (entry **1**) in the remotely controlled synthesis module. The activity was eluted with an aqueous solution of K_2_CO_3_ (1.78 mg/0.4 mL water; entry **2**), mixed with K_2.2.2_ (11.2 mg/1 mL MeCN; entry **3**) into the reaction vessel (entry **6**) and azeotropically dried for approximately 10 min. Thereafter, 1 mg of the tosylate **TA3a** dissolved in 1 mL MeCN (entry **4**) was added, and the reaction mixture was stirred at 80 °C for 15 min. After cooling, the reaction mixture was diluted with 2 mL water (entry **5**) and transferred into the injection vial. Semi-preparative HPLC (system **A**, entry **7**) was performed for isolation of the desired radioligand [^18^F]**TA3** (*t_R_* = 27–29 min). [^18^F]**TA3** was collected in entry **8** previously loaded with 40 mL water. Final purification step took place after passing the solution through a Sep-Pak^®^ C18 Plus light cartridge (Waters; pre-conditioned with 20 mL of absolute EtOH and 60 mL water; entry **11**), followed by washing with 2 mL water (entry **9**) and elution of [^18^F]**TA3** with 1 mL of absolute EtOH (entry **10**) into the product vial (entry **12**).

To obtain an injectable solution, the solvent was reduced under a gentle nitrogen stream at 70 °C and the radioligand was formulated in sterile isotonic saline containing 10% EtOH (*v*/*v*). Analytical radio-HPLC (system **B**, gradient and isocratic mode) of the final product spiked with the non-radioactive reference compound **TA3** confirmed the identity of [^18^F]**TA3**.

### 3.5. Determination of Lipophilicity (logD_7.4_) and in Vitro Stability

The lipophilicity of [^18^F]**TA3** or [^18^F]**TA4** was determined by partitioning between *n*-octanol and phosphate buffered saline (PBS, pH 7.4) at ambient temperature using the conventional shake-flask method. An aliquot of 10 µL of the formulated solutions of each [^18^F]**TA3** or [^18^F]**TA4** with approximately 1 MBq of the radioligand was added to a tube containing 6 mL of the *n*-octanol/PBS-mixture (1:1, *v*/*v*, fourfold determination). The tubes were shaken for 20 min using a mechanical shaker (HS250 basic, IKA Labortechnik GmbH & Co. KG, Staufen, Germany) followed by centrifugation (5000 rpm for 5 min) and separation of the phases. Aliquots of each 1 mL were taken from the organic and the aqueous phase and activity was measured using an automated gamma counter (1480 WIZARD, Fa. Perkin Elmer, Waltham, MA, USA). The distribution coefficient (D) was calculated as [activity (cpm/mL) in *n*-octanol]/[activity (cpm/mL) in PBS, pH 7.4] specified as the decade logarithm (logD_7.4_).

*In vitro* stabilities of [^18^F]**TA3** or [^18^F]**TA4** were investigated by incubation of the radioligands in phosphate buffered saline (PBS, pH 7.4), *n*-octanol and pig plasma at 37 °C for 60 min (~5 MBq of the radioligand added to 500 µL of each medium). Samples were taken at 15, 30 and 60 min after incubation and analyzed by radio-TLC and radio-HPLC (system **B**).

### 3.6. Animal Studies

All animal procedures were approved by the Animal Care and Use Committee of Saxony (TVV 08/13).

#### 3.6.1. *In Vitro* Autoradiographic Studies in Rat Brain

Frozen sagittal brain sections obtained from female SPRD rats (10–12 weeks old) were thawed, dried in a stream of cold air, and preincubated for 20 min with incubation buffer (50 mM TRIS-HCl, pH 7.4, 120 mM NaCl, 5 mM KCl, 2 mM CaCl_2_, 5 mM MgCl_2_) at ambient temperature. Brain sections were incubated with ~1 MBq/mL of [^18^F]**TA3** or [^18^F]**TA4** in incubation buffer for 60 min at ambient temperature. Afterwards sections were washed twice with 50 mM TRIS-HCl (pH 7.4) for 2 min at 4 °C, dipped briefly in ice-cold deionized water, dried in a stream of cold air and exposed for 60 min to an ^18^F-sensitive image plate that was analyzed afterwards using an image plate scanner (HD-CR 35; Duerr NDT GmbH, Bietigheim Bissingen, Germany). Nonspecific binding of the radioligand under investigation was determined by co-incubation with 10 µM **TA1**.

#### 3.6.2. Small-Animal PET/MR Studies in Mice

Female CD-1 mice (*n* = 4; age: 8 weeks; weight: 28.8 ± 1.2 g; supplier: Medizinisch-Experimentelles Zentrum Leipzig, Leipzig, Germany) were housed under a 12 h:12 h light-dark cycle at 26 °C in a vented animal cabinet. The animals received an injection of 9.7 ± 1.3 MBq of [^18^F]**TA3** into the tail vein followed by a 60 min PET/MR scan (Mediso nanoScan^®^, Budapest, Hungary). Each PET image was corrected for random coincidences, dead time, scatter and attenuation (AC), based on a whole body (WB) MR scan.

The reconstruction parameters for the list mode data are: 3D-ordered subset expectation maximization (OSEM), 4 iterations, 6 subsets, energy window: 400–600 keV, coincidence mode: 1–5, ring difference: 81. The mice were positioned prone in a special mouse bed (heated up to 37 °C), with the head fixed to a mouth piece for the anesthetic gas supply with isoflurane in 40% air and 60% oxygen (anesthesia unit: U-410, agnthos, Lidingö, Sweden; Gas blender: MCQ, Rome, Italy). The PET data was collected by a continuous WB scan during the entire investigation. Following the 60 min PET scan a T1 weighted WB gradient echo sequence (GRE, T_R_ = 20 ms; T_E_ = 6.4 ms) was performed for AC and anatomical orientation. Image registration and evaluation of the region of interest (ROI) was done with ROVER (ABX advanced biochemical compounds, Radeberg, Germany).

The respective brain regions were identified using the MR information from the GRE scan. The activity data is expressed as mean standardized uptake value (SUV) of the overall ROI.

#### 3.6.3. *In Vivo* Metabolism Studies in Mice

The radioligands [^18^F]**TA3** or [^18^F]**TA4** (~60 MBq in 150 µL isotonic saline) were injected via the tail vein in female CD-1 mice (10–12 weeks old). Brain and blood samples were obtained at 30 min p.i., plasma separated by centrifugation (14,000× *g*, 1 min), and brain homogenized in ~1 mL isotonic saline on ice (10 strokes of a PTFE plunge at 1000 rpm in a borosilicate glass cylinder; Potter S Homogenizer, B. Braun Melsungen AG, Melsungen, Germany).

### 3.7. Conventional Extraction Procedure

Twofold extractions of plasma (2 × 50 µL, 30 min p.i.) and brain samples (2 × 250 µL, 30 min p.i.) were performed using a mixture of ice-cold acetone/water (8:2, *v*/*v*; plasma or brain sample/organic solvent, 1:4, *v*/*v*). The samples were vortexed for 1 min, incubated on ice for 10 min (first extraction) or 5 min (second extraction) and centrifuged at 10,000 rpm for 5 min. Supernatants were collected and the precipitates were re-dissolved in ice-cold acetone/water for the second extraction. Aliquots from supernatants of each extraction step were taken. Activity thereof and of the precipitates was quantified using an automated gamma counter (1480 WIZARD, Fa. Perkin Elmer). The supernatants from both extractions were combined, concentrated under nitrogen stream at 70 °C and analyzed by radio-HPLC (system **B**).

### 3.8. Micellar Chromatography (MLC)

For preparation of the MLC injection samples, mouse plasma (20–50 µL, 30 min p.i.) was dissolved in 100–300 µL of 200 mM aqueous SDS and injected directly into the MLC system (500 µL sample loop; column: Reprosil-Pur C18-AQ, 250 × 4.6 mm, particle size: 10 µm; gradient: 0–15 min: 3% 1-PrOH, 15–40 min: 3% → 30% 1-PrOH; 40–49 min: 30% 1-PrOH, 49–50 min: 30% → 3% 1-PrOH; 50–60 min: 3% 1-PrOH/100 mM SDS, 10 mM Na_2_HPO_4_; flow: 1 mL/min; ambient temperature). Notably, a pre-column with 10 mm length was used and frequently exchanged to expand the life time of the RP-column. Homogenized brain material (100–200 µL, 30 min p.i.) was dissolved in 500 µL of 200 mM aqueous SDS, stirred at 75 °C for 5 min and after cooling to ambient temperature injected into the MLC system.

## 4. Conclusions

In conclusion, the novel radioligands [^18^F]**TA3** and [^18^F]**TA4** are well suitable for *in vitro* imaging of PDE2A but further structural modification might prevent the formation of brain penetrating radiometabolites. Thus, these modifications could lead to ^18^F-radioligands enabling *in vivo* PET imaging of PDE2A in brain.

Furthermore, compared to the conventional extraction procedure combined with analytical RP-HPLC, it could be shown that micellar HPLC is a more reliable method to quantify the amount of non-metabolized [^18^F]**TA3** and [^18^F]**TA4** not only in plasma samples but also in brain homogenates.
